# Roles of cerebrospinal fluid-contacting neurons as potential neural stem cells in the repair and regeneration of spinal cord injuries

**DOI:** 10.3389/fcell.2024.1426395

**Published:** 2024-06-25

**Authors:** Yanxiang Xiong, Wenjun Pi, Wang Zhao, Weiwei Shi, Weihong Yan, Hao Yang, Yuanrong Zhou, Qing Li, Leiluo Yang

**Affiliations:** ^1^ Department of Traumatic Orthopedics, The Affiliated Hospital of Guizhou Medical University, Guiyang, Guizhou, China; ^2^ Department of Medical Examination Center, The Affiliated Hospital of Guizhou Medical University, Guiyang, Guizhou, China; ^3^ Department of Health, The Qinglong County People’s Hospital, Qinglong, Guizhou, China

**Keywords:** neural stem cells, neural regeneration, CSF-cNs, spinal cord injury, repair, PKD2L1

## Abstract

Cerebrospinal fluid-contacting neurons (CSF-cNs) represent a distinct group of interneurons characterized by their prominent apical globular protrusions penetrating the spinal cord’s central canal and their basal axons extending towards adjacent cells. Identified nearly a century back, the specific roles and attributes of CSF-cNs have just started to emerge due to the historical lack of definitive markers. Recent findings have confirmed that CSF-cNs expressing PKD2L1 possess attributes of neural stem cells, suggesting a critical function in the regeneration processes following spinal cord injuries. This review aims to elucidate the molecular markers of CSF-cNs as potential neural stem cells during spinal cord development and assess their roles post-spinal cord injury, with an emphasis on their potential therapeutic implications for spinal cord repair.

## 1 Introduction

CSF-cNs, a distinct neuronal class with direct connections to cerebrospinal fluid (CSF), were first depicted by Kolmer and Agduhr (Kolmer, 1921; Agduhr, 1922). Subsequently, Vign et al. investigated the unique morphology of CSF-cNs using electron microscopy and described CSF-cNs in most vertebrate spinal cords (Vigh-Teichmann and VIGH, 1983a). According to the report, dendritic terminals of CSF-cNs extend into the spinal cord’s central canal (Dale et al., 1987a; Vigh and Vigh‐Teichmann, 1998). Further research demonstrated that, in some species, the terminals possess multiple microvilli (Vigh et al., 1983; Böhm et al., 2016), or motile cilia (Vigh and Vigh-Teichmann, 1973; Vigh-Teichmann and Vigh, 1983b; Vígh et al., 2004).

Spinal cord injury (SCI) is an intractable and highly disabling condition, lacking effective therapeutic strategy (Tohda and Kuboyama, 2011). Recent advances have positioned neural stem cell (NSC) transplantation as a focal point in spinal cord injury treatment. However, the outcomes of transplantation vary widely (Reekmans et al., 2012; Gao et al., 2013; Huang et al., 2022). There remains a substantial scientific debate concerning the origin of NSCs in the spinal cord of mammals (Teng et al., 2008; Finkel et al., 2021). Recent studies confirmed that CSF-cNs possess NSC properties (Wang et al., 2021a), positioning them as critical for endogenous spinal cord repair.

In this review, we outline the primary biomarkers of CSF-cNs and their pivotal role in spinal cord injury therapeutic application. Cao et al. (2022) have identified CSF-cNs as possessing neural stem cell (NSC) potential, marked by their ability to form neurospheres and express NSC markers, suggesting a role in neural regeneration and repair post-spinal cord injury. Importantly, CSF-cNs exhibit multipotent differentiation capabilities; they can develop into neurons, astrocytes, and oligodendrocytes *in vitro*. This capacity not only underscores the versatile potential of CSF-cNs in contributing to neural regeneration but also opens new therapeutic avenues for spinal cord injury treatment. The discovery of CSF-cNs as a novel NSC type enriches our understanding of neuroregeneration mechanisms within the spinal cord, and aids in the development of innovative strategies for enhancing functional recovery post-SCI. By elucidating the characteristics and mechanisms of CSF-cNs, this research provides a foundation for improving rehabilitation techniques and treatment outcomes for spinal cord injury patients (Cao et al., 2022).

## 2 The molecular markers of cerebrospinal fluid contacting neurons

For decades, research on CSF-cNs was impeded due to the absence of distinct markers. In 2006, Huang et al. (2006) advanced this field by identifying PKD2L1 channels as specific markers for CSF-cNs. Further, transcription factors like GATA2 and GATA3 could help to indentify CSF-cNs (Petracca et al., 2016a; Andrzejczuk et al., 2018). Moreover, the detection of GABA, acid-sensing ion channels (ASICs), and various neuromodulators in CSF-cNs has broadened the understanding of their properties and functions (Dale et al., 1987c).

### 2.1 Cerebrospinal fluid-contacting neurons and PKD2L1

CSF-cNs in taste buds responsible for sour tastedetection express PKD2L1 (Huang et al., 2006), and this channel is typically involved in the detection of chemical, thermal, and mechanical stimuli (Retailleau and Duprat, 2014; Hussein, 2015). Among the seven subfamilies of TRPs, one well-known channel, PKD2L1, belongs to the transient receptor potential polycystin (TRPP) subfamily, which includes polycystic kidney disease (PKD) proteins (Vígh et al., 2004; Orts-Del’immagine et al., 2012a). Notably, the PKD2L1 channel remains consistent in CSF-cNs in the spinal cord (Djenoune et al., 2014). CSF-cNs selectively express PKD2L1 (Orts-Del'Immagine et al., 2016a; Orts-Del’Immagine et al., 2014a; Orts-Del’Immagine et al., 2017), which is now widely considered a specific marker for CSF-cNs (Wyart et al., 2023). When extracellular or intracellular calcium levels are elevated, or in hypotonic conditions, this channel facilitates large current of CSF-cNs (Orts-Del'Immagine et al., 2016a; Jurčić et al., 2019). PKD2L1 is capable of generating significant depolarizations, acting as a spike generator that can initiate action potentials (Orts-Del'Immagine et al., 2016a; Sternberg et al., 2018a). Additionally, PKD1L2 and PKD2L1 co-expression in CSF-cNs suggests a regulatory interaction (Petracca et al., 2016b; England et al., 2017), moderating neuronal excitation by influencing PKD2L1’s membrane localization (Orts-Del'Immagine et al., 2016b). This elucidates the physiological roles of CSF-cNs, guided by the pivotal discovery of PKD2L1 (Orts-Del'Immagine and Wyart, 2017).

### 2.2 Cerebrospinal fluid-contacting neurons and GABA

γ-aminobutyric acid (GABA) serves as the primary inhibitory neurotransmitter in the CNS (Roberts, 1974; Watanabe et al., 2002), transitioning from excitatory to inhibitory effects during neuronal maturation (Ben-Ari, 2014). GABA expression in CSF-cNs has been observed across various species (Dale et al., 1987b; Feldblum et al., 1995; Binor and Heathcote, 2001; Yang et al., 2010). Dale et al. described a specific CSF-cN subclass in the spinal cords of frog embryos, located ventrolaterally (Dale et al., 1987c; Dale et al., 1987d). Detailed anatomical studies on CSF-cNs, including their axonal projections, developmental stages, and distribution in amphibians and zebrafish, have employed anti-GABA and glutamic acid decarboxylase (GAD) antibodies for accurate immunolabeling (Bernhardt et al., 1992; Binor and Heathcote, 2001). These neurons are classified as GABAergic through immunohistochemical techniques (Djenoune et al., 2014; Djenoune and Wyart, 2017a). The identification of CSF-cNs primarily depends on their GABAergic properties, proximity to the central canal, and lumen contact (Dale et al., 1987c).

### 2.3 Cerebrospinal fluid-contacting neurons and transcription factors

Transcription factors are pivotal in the generation of CSF-cNs. Ascl1, a basic helix-loop-helix (bHLH) transcription factor, governs the balance between ependymal cells and CSF-cNs in the spinal cord’s central canal. It is notably expressed in the precursor cells of CSF-cNs, facilitating proper cell population distribution (Di Bella et al., 2019a). Nkx6.1, another transcription factor, promotes the differentiation of motoneurons, with PKD2L1^+^ CSF-cNs express Nkx6.1 (Orts-Del'Immagine et al., 2014a). Additionally, The co-expression of Nkx6.1 and Pax6 serves as a marker for CSF-cNs (Petracca et al., 2016b). Other significant transcription factors, Nkx2.2 and Foxa2, are involved in embryonic development and differentiation and also mark CSF-cNs (Di Bella et al., 2019b). Although PKD2L1 has traditionally been a primary identifier for CSF-cNs (Djenoune et al., 2014; Djenoune et al., 2017a; Sternberg et al., 2018a), the specific combination of PKD2L1 with GATA2 and GATA3 has been proposed as a more comprehensive marker (Petracca et al., 2016b). These transcription factors have been consistently used in recent studies for identifying CSF-cNs (Di Bella et al., 2019a), which may enhance future research methodologies.

### 2.4 Cerebrospinal fluid-contacting neurons and ASIC

Acid-base homeostasis is essential for organism survival, with ASICs playing a pivotal role as proton-gated excitatory cation channels responsive to pH changes (Boscardin et al., 2016; Soto et al., 2018). ASIC3, the most widely expressed subtype in sensory neurons and nerve terminals, translates mechanical and nociceptive stimuli into electrical signals (Chen and Wong, 2013). Present both peripherally and in the CNS (Deval and Lingueglia, 2015), ASIC3 functions as an electrochemical sensor for somatic and visceral nociception (Leppert et al., 2016). Peripherally, it is essential for managing inflammatory pain and modulating pain signals, including mechano-sensation, at the spinal cord level (Zha, 2013). CSF-cNs, expressing ASIC3, respond to both mechanical stimulation and pH variations (Jalalvand et al., 2016a), with their acidic response and fluid-induced mechanical reactions mediated by ASIC3 channels (Jalalvand et al., 2016b; Jalalvand et al., 2018).

### 2.5 Cerebrospinal fluid contacting neurons and neuropeptides

Neuropeptides critically regulate CSF-cNs, maintaining neural function and nervous system health (Smith et al., 2004). CSF-cNs serve as production sites for neuropeptides Urp1 and Urp2 (Quan et al., 2015; Zhang et al., 2018; Gaillard et al., 2023), primarily co-expressed in zebrafish spinal cords (Quan et al., 2015). Additionally, somatostatin 1.1 (sst1.1) is uniquely present in the dorsal CSF-cNs of zebrafish, marking this specific subset (Djenoune et al., 2017b). Neuropeptide C (Nppc), urocortin 3 (Ucn3), and tachykinin 3 (Tac3) also serve as biomarkers for CSF-cNs in zebrafish (Wilson et al., 2017; Prendergast et al., 2023). In contrast, in macaque monkeys, the vasoactive intestinal peptide (VIP) is a distinct marker for CSF-cNs (LaMotte, 1987).

### 2.6 Cerebrospinal fluid-contacting neurons and secreted proteins

Secretory proteins from CSF-cNs are integral for modulating neuronal activity and enhancing communication (Vígh et al., 2004; Orts-Del’immagine et al., 2012a). Notably, prostate-associated microseminoprotein (Msmp) is recognized as a potential biomarker for CSF-cNs (Prendergast et al., 2023). Additionally, secretogranin-2 (Scg2), important in neurosecretion (Wyart et al., 2023), and the enzyme aromatic L-amino acid decarboxylase (AADC), which synthesizes trace amines, are expressed in these neurons (Wyart et al., 2023). In conclusion, CSF-cNs exhibit distinct features crucial for spinal cord repair, including prominent apical protrusions that facilitate cellular interactions. Cao et al. (2022) highlight the expression of PKD2L1 in CSF-cNs, suggesting their role as neural stem cells aiding neuroregeneration post-SCI and their involvement in maintaining cellular structure, connectivity, and internal regulation.

### 2.7 Cerebrospinal fluid-contacting neurons and monoamines

Monoamines have a substantial impact on the modulating the excitatory and inhibitory of CSF-cNs (Djenoune and Wyart, 2017a). Dopamine, a key monoamine, is essential for the functional regulation of CSF-cNs (Orts-Del’Immagine et al., 2014b). Studies conducted previously have documented the synthesis of dopamine by ventral CSF-cNs in lampreys (Wyart et al., 2023). Additionally, transient expression of serotonin in CSF-cNs of fish (Montgomery et al., 2016; Djenoune et al., 2017b) and birds (Vígh et al., 2004; Orts-Del’immagine et al., 2012a) has been observed, with the biosynthesis mediated by tryptophan hydroxylase 2 (TPH2) (Hirunagi et al., 1992).

### 2.8 Cerebrospinal fluid-contacting neurons and structural proteins

Structural proteins such as Myo3b and spin are crucial for maintaining cellular morphology and are specifically expressed in the distal areas of CSF-cNs within the spinal cord (Desban, 2018; Desban et al., 2019). Moreover, flagella-associated protein 57 (FAP57) not only is expressed in CSF-cNs, but also serves as an effective biomarker for identifying CSF-cNs (Wyart et al., 2023). Hence, CSF-cNs are distinctive in their role in spinal cord repair, featuring apical bulbous protrusions that facilitate cellular connections through the central canal. Cao et al. highlight that CSF-cNs express PKD2L1, attributing them with neural stem cell-like properties that aid neuroregeneration post-injury. These neurons also play key roles in sustaining cellular structure, enhancing connectivity, and regulating intracellular activities.

## 3 Neural stem cell potential of cerebrospinal fluid-contacting neurons

In the mammalian brain, neural stem cells (NSCs) are found in three main regions: the lateral ventricles’ subventricular zone (SVZ), the hippocampus’ subgranular zone (SGZ), and within the spinal cord’s central canal, all supported by growing evidence (Stoeckel ME. et al., 2003; Sabourin et al., 2009; Chaker et al., 2016; Djenoune and Wyart, 2017b). In the spinal cord’s central canal, three distinct cell types can be identified based on morphological characteristics: ciliated ependymal cells with short basal processes that interface with adjacent neural tissue, mono-ciliated ependymal cells, and CSF-cNs (Song and Zhang, 2018; Moreno-Manzano, 2020). Historically, ependymal cells were thought to be NSCs in the spinal cord (Meletis et al., 2008; Marichal et al., 2012; Ortiz-Álvarez et al., 2019; Redmond et al., 2019; Moreno-Manzano, 2020). However, recent single-cell RNA sequencing shows that although they express genes related to cilia, they also exhibit multiple stem cell-associated genes (Ortiz-Alvarez et al., 2019), yet lack typical NSC functions under stimulatory conditions *in vivo* and *in vitro* (Shah et al., 2018; Xue et al., 2021).

CSF-cNs, prominently located in the central canal of the spinal cord and other brain regions such as the dorsal raphe nuclei and hypothalamus (Tonelli Gombalova et al., 2020; Yang et al., 2020; Jurcic et al., 2021), begin to develop from the 12th day of embryonic life, peaking around days E14 and E15. Unlike other spinal neurons, CSF-cNs are not restricted to specific neuroepithelial regions and can arise during later neurogenic phases (Kutna et al., 2014).

The somata of cerebrospinal fluid-contacting neurons (CSF-cNs) are located near the central canal of the spinal cord, a site of neurogenic potential in adult vertebrates capable of endogenous repair (Becker et al., 2018). Electrophysiological studies on CSF-cNs *in vitro* (Orts-Del'Immagine et al., 2016a; Orts-Del’immagine et al., 2012b; Petracca et al., 2016c; Reali et al., 2011) and *in vivo* (Sternberg et al., 2018b) have consistently shown elevated membrane resistance (Orts-Del’immagine et al., 2012b; Sternberg et al., 2018b). Immature neurons usually possess a very high membrane resistance. Consequently, spinal CSF-cNs display numerous molecular indicators indicative of neurons development, including reduced NeuN expression and increased levels of doublecortin (DCX) (Ren et al., 2017; Prendergast et al., 2023), homeobox protein NKX6.1, ELAV-like protein 3 (ELAVL3), Ascl1 (Di Bella et al., 2019c) and the polysialylated form of the neural cell adhesion molecule (PSA-NCAM) (Orts-Del'Immagine et al., 2014b; Reali et al., 2011; Stoeckel M-E. et al., 2003; Kútna et al., 2014; Marichal et al., 2009; Russo et al., 2004). These expression patterns indicate a sustained immature state in CSF-cNs, which may enhance their structural flexibility and regenerative capacity following injuries (Bonfanti and Seki, 2021).

Mouse PKD2L1-positive CSF-cNs neurospheres cultivated *in vitro* exhibit NSC markers expression, proliferative ability, and potential for differentiation into neurons, and oligodendrocytes (Cao et al., 2023). Further *in vivo* studies indicated that spinal cord injuries or the injection of neurotrophic factors such as bFGF and VEGF into the lateral ventricle can activate and enhance the proliferation of CSF-cNs (Cao et al., 2022). CSF-cNs were also found to express aromatic amino acid decarboxylase (AADC) in response to injury, facilitating monoamines synthesis (Wienecke et al., 2014; Ren et al., 2016; Ren et al., 2017). Remarkably, evidence of recovery following SCI has shown substantial resilience in CSF-cNs, particularly in those concentrated in the lumbar region of the spinal cord in mice, which regained ambulatory abilities through electrical stimulation therapy (Kathe et al., 2022). Additionally, CSF-cNs are abundantly present in the mature primate spinal cord (LaMotte, 1987; Djenoune et al., 2014; Kastner and Wanaverbecq, 2023). These findings collectively highlight the significant NSC potential of CSF-cNs. Gerstmann et al. (2022) posited that cerebrospinal fluid-contacting neurons (CSF-cNs) are integral to mammalian spinal cord motor control, particularly in adaptive control and proprioceptive feedback. These neurons contribute to motor regulation and limb movement fine-tuning by interfacing with the spinal motor circuit and monitoring cerebrospinal fluid dynamics. However, *in vivo* studies on the stem cell properties of CSF-cNs are still limited, focusing mainly on their potential differentiation capabilities with insufficient exploration of specific signaling pathways and regulatory factors. Additionally, the impact of post-SCI microenvironments, such as inflammatory and immune responses, on CSF-cNs is under-researched. It is essential to conduct comprehensive studies on these aspects to better understand CSF-cNs’ differentiation mechanisms and their stem cell functionality post-SCI, which could advance their clinical applications.

## 4 Neural stem cells regeneration in spinal cord injuries

**FIGURE 1:**
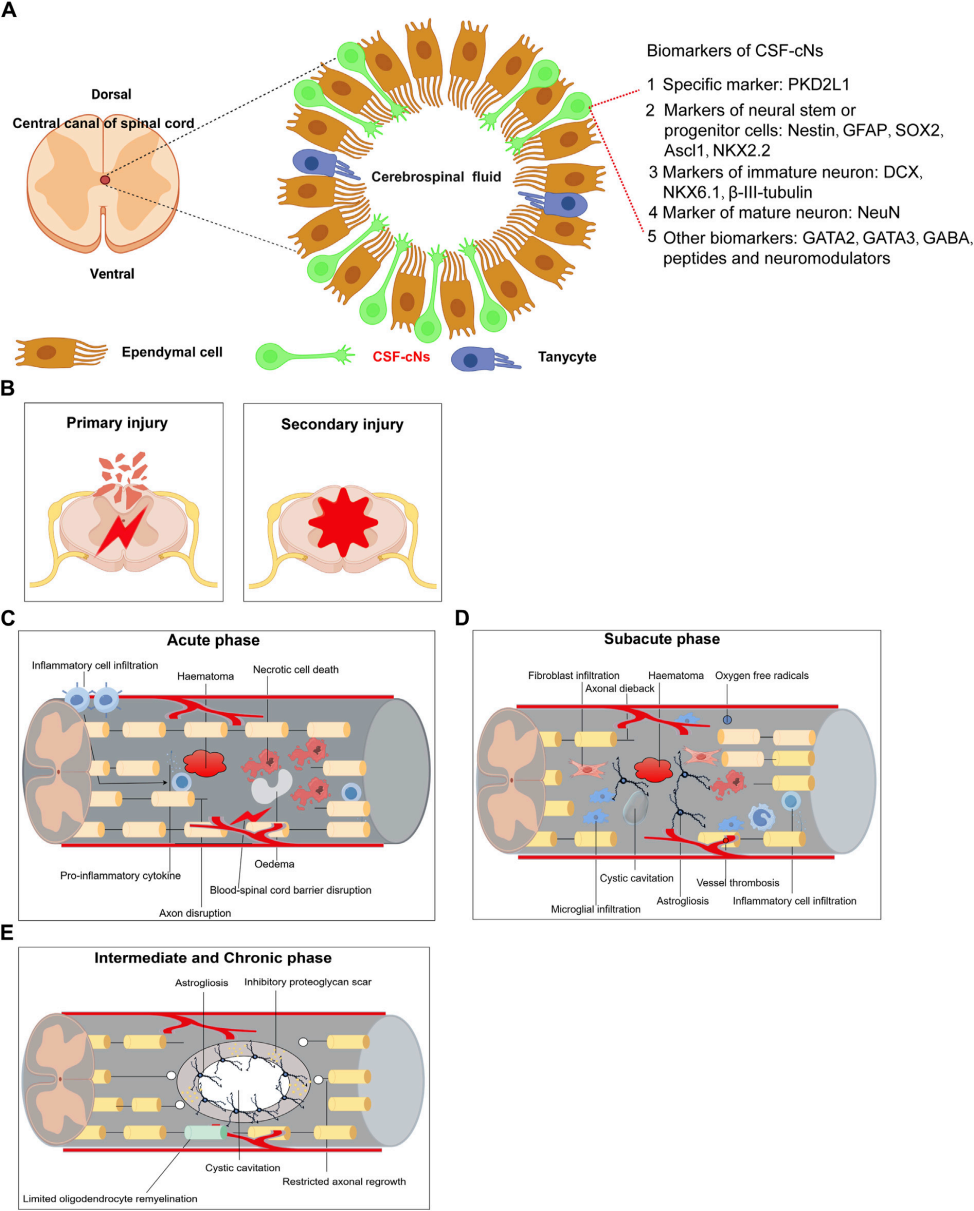
Summarizes the main biomarkers of CSF-cNs and Pathological mechanisms of spinal cord injury. **(A)** Summarizes the main biomarkers of CSF-cNs. **(B)** Primary Injury and Secondary Injury of spinal cord injury. **(C)** Acute phase of spinal cord injury. **(D)** Subacute phase of spinal cord injury. **(E)** Intermediate and Chronic phase of spinal cord injury.

**FIGURE 2:**
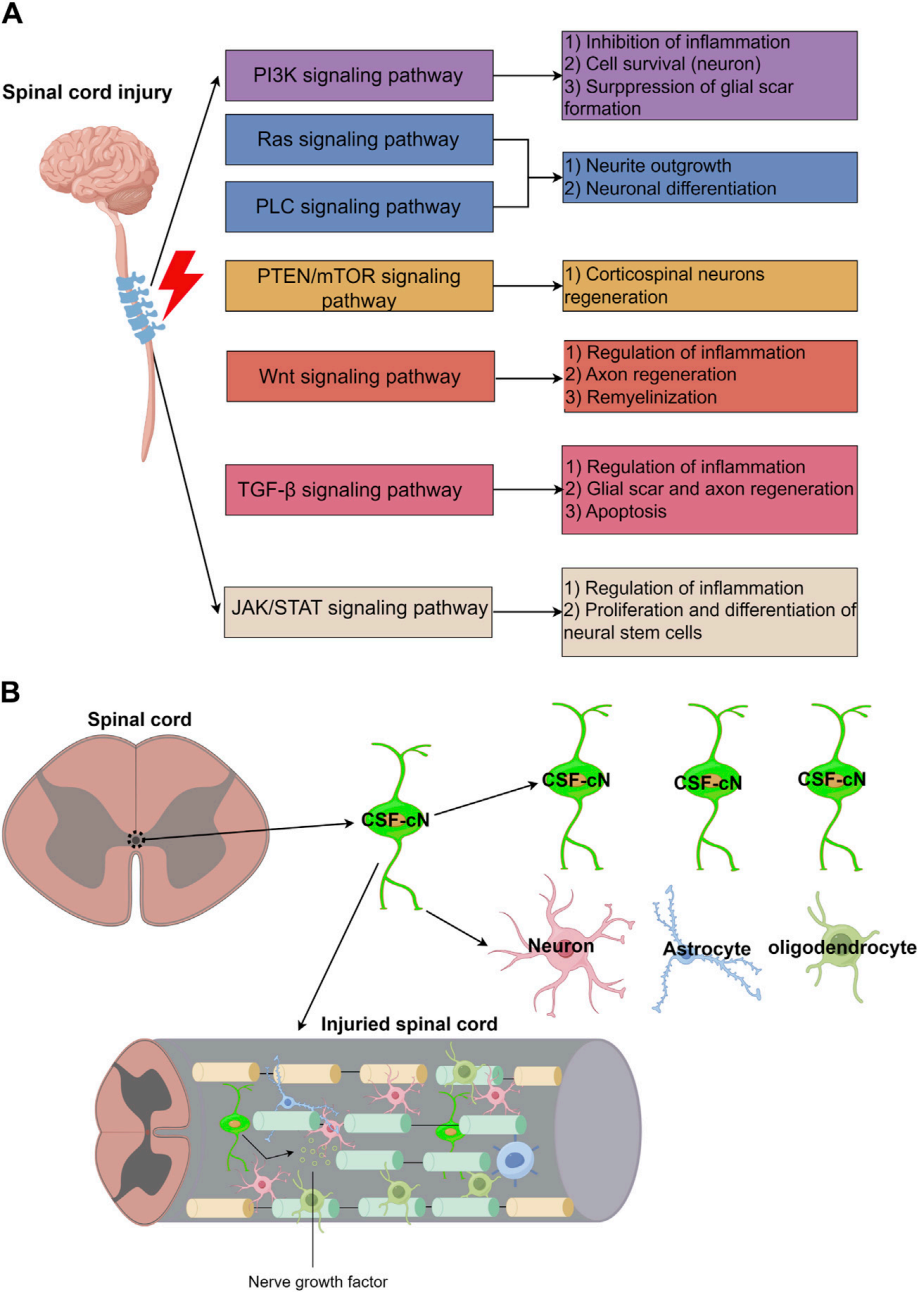
The main signaling pathways in spinal cord injury and the main mechanism of CSF-cNs in repair after spinal cord injury. **(A)** Main signaling pathways in spinal cord injury. **(B)** Main mechanism of CSF-cNs in repair after spinal cord injury.

After spinal cord injury in rats, a decline in endogenous neural progenitor cell populations and locomotor function was observed, underscoring the potential role of CSF-cNs in post-injury neurological functions (He et al., 2020). Immunofluorescence studies showed that neurospheres derived from PKD2L1+ CSF-cNs expressed neural stem cell markers such as Sox2, Nestin, and GFAP (Cao et al., 2023), and were capable of differentiating into neurons, oligodendrocytes, and astrocytes *in vitro* (He et al., 2020). These findings suggest that CSF-cNs are immature neuronal cells with the properties of neural stem cells, able to proliferate and differentiate into various neuronal types (Wang et al., 2021b). Additionally, the significant recovery noted post-injury highlights the robust regeneration capacity of rodent CSF-cNs, which support locomotor recovery and restore ambulatory function via electrical stimulation in the lumbar spinal cord (Kathe et al., 2022). Figure 1A outlines the main biomarkers of CSF-cNs. Spinal cord injury (SCI) involves complex pathological processes, categorized into primary and secondary injuries (Figure 1B). The acute phase is characterized by inflammation, hematoma formation, neuronal death, and blood-spinal cord barrier disruptions (Figure 1C). The subacute phase features fibroblast and microglial infiltration, cyst formation, and axonal dieback (Figure 1D), while the intermediate and chronic phases are marked by astrogliosis, limited remyelination, and restricted axonal regrowth (Figure 1E). This article also examines signaling pathways influencing SCI repair, including PI3K/Akt, Ras, PLC, PTEN/mTOR, Wnt, and TGF-β, which regulate cell survival, inflammation, neurite outgrowth, and axon regeneration (Figure 2A). JAK/STAT pathway’s role in mediating inflammation and supporting neural stem cell functions is also highlighted (Figure 2A). CSF-cNs contribute significantly to SCI repair by differentiating into neurons, astrocytes, and oligodendrocytes, enhancing neural repair and supporting nerve function (Figure 2B). Neurons transmit electrical signals, supported by neurotrophic factors for survival and regeneration. Astrocytes provide metabolic support and regulate inflammation, while oligodendrocytes maintain myelin sheaths, crucial for efficient nerve impulse transmission.

The aforementioned findings indicate that CSF-cNs in the spinal cord exhibit NSCs properties. Furthermore, NSCs derived from CSF-cNs in the central canal may serve as a reservoir of neuronal cells, aiding in the replenishment of the damaged region (Wang et al., 2021a). Despite advancements in treatment, SCI often results in significant neurological deficits. The discovery of adult NSCs in mammals, including humans, has enhanced the prospects for treating CNS disorders by promoting self-regeneration (Chaker et al., 2016; Moore, 2016). Conversely, the inherent stem cell properties of CSF-cNs may revolutionize therapeutic approaches for patients with spinal cord injury, suggesting that CSF-cNs play a pivotal role in the treatment of SCI. Future research should focus on the molecular mechanisms of CSF-cNs, particularly factors influencing their neural stem cell characteristics, proliferation, and differentiation. Understanding these mechanisms could identify new targets for developing precise SCI treatment strategies. Exploring the use of CSF-cNs in stem cell therapy might offer avenues for enhancing neural regeneration and functional recovery post-SCI. Additionally, investigations into pharmacological interventions that modulate CSF-cNs’ growth and differentiation could lead to novel therapeutic approaches for SCI. The discovery of regulatory drugs or growth factors may facilitate new drug-based strategies. The potential of CSF-cNs research in clinical therapy includes innovating treatment methods and enhancing the quality of life for SCI patients through personalized, condition-specific therapies that minimize treatment risks and optimize outcomes.

## 5 Perspectives

Currently, the scientific community is increasingly focusing on the role of CSF-cNs, particularly in their potential for addressing spinal cord pathologies. The intimate association of these neurons with CSF makes intrathecal therapy a viable approach. Despite considerable progress in the characterization of CSF-cNs, highlighted by the identification of the unique marker PKD2L1, many aspects of their neural stem cell-like properties *in vitro* or *in vivo* remain poorly understood. For example, of the two distinct CSF-cN populations identified, which one retains neural stem cell properties? In SCI repair, Wyart et al. (2023) considered dorsal CSF-cNs may have closer contact with the CSF, potentially being involved in sensory signal transmission, the restoration of sensory function, and more prominently in posture control and stability. On the other hand, ventral CSF-cNs may be more connected to spinal cord tissue, potentially participating in motor signal transmission, the restoration of motor function, and more prominently in motor control and rapid swimming. These distinct locations and functions may allow CSF-cNs to synergistically contribute to SCI repair, promoting the recovery of neural function and the regeneration of nerve cells. Moreover, the processes by which CSF-cNs act as neural stem cells in spinal cord injury conditions and their role in lesion repair are still to be fully elucidated. Future research should continue to explore the activation and differentiation of CSF-cNs in spinal cord injury scenarios and aim to clarify the specific roles these cells play post-spinal cord injury.
